# Supplementary material for: A Survey on Undergraduate Medical Students’ Perception of COVID-19 Vaccination
Source: Vaccines (Basel). 2022 Sep 3;10(9):1464. doi: 10.3390/vaccines10091464 (PMC9504072; doi:10.3390/vaccines10091464)
Supplement: Supplementary file 1 [file vaccines-10-01464-s001.zip › vaccines-1863882-supplementary.pdf]

Socio-demographic characteristics

Gender:

- ☐ Male
- ☐ Female
- ☐ I prefer not to specify it

Age (years):

☐

Nationality:

- ☐ Italian
- ☐ Other

If you answered other, please specify your nationality:

☐

County where you lived permanently at the beginning of the pandemic (March 2020):

☐

Housing modality:

- ☐ Alone
- ☐ With family members
- ☐ With friends
- ☐ With cohabitant
- ☐ I prefer not to answer

Characteristics relating to the state of health

Have you contracted SARS-CoV-2 disease?

- ☐ Yes, without the development of symptoms or with mild to moderate symptoms
- ☐ Yes, with hospitalization
- ☐ No
- ☐ I prefer not to answer

Do you suffer from a chronic disease?

- ☐ Yes
- ☐ No

Have you already undergone or will you undergo the COVID-19 vaccine?

- Yes
- No, but I plan to do it as soon as possible
- No
- I don't know

Answer only if you have undergone the COVID-19 vaccination

What was the motivation for getting the COVID vaccine? (It is possible to indicate more than one answer):

- To protect myself
- To protect my family
- For moral responsibility towards the community
- To get the green pass
- Other

Indicate other reasons:

Answer only if you have not undergone the COVID-19 vaccination

What was your motivation NOT to undergo the COVID vaccine? (It is possible to indicate more than one answer):

- Fear of vaccines in general
- Fear of this vaccine
- Insufficient and hasty experimentation
- Contradictory information on efficacy and safety
- Ineffectiveness of the vaccine
- Sense of compulsion to vaccinate
- The belief that the vaccination campaign is economic or other propaganda

Indicates the degree of agreement where 0 is "Not at all", 10 "very much" of agreement

COVID-19 vaccines have been adequately tested

0 1 2 3 4 5 6 7 8 9 110

COVID-19 vaccines represent an indispensable tool for the protection of individual and public health

0 1 2 3 4 5 6 7 8 9 110

Vaccination against COVID-19 represents an ethical duty for the entire community to protect individual and public health

☒ 0 ☐ 1 ☐ 2 ☐ 3 ☐ 4 ☐ 5 ☐ 6 ☐ 7 ☐ 8 ☐ 9 ☐ 10

It is essential to impose a COVID-19 vaccination obligation on the whole community to protect the fragile population

☒ 0 ☐ 1 ☐ 2 ☐ 3 ☐ 4 ☐ 5 ☐ 6 ☐ 7 ☐ 8 ☐ 9 ☐ 10

It is essential to impose a COVID-19 vaccination obligation on all university students to protect people who cannot access the vaccine for medical reasons

☒ 0 ☐ 1 ☐ 2 ☐ 3 ☐ 4 ☐ 5 ☐ 6 ☐ 7 ☐ 8 ☐ 9 ☐ 10

It is appropriate to exclude the access to the university of teachers / students who, although not presenting medical-health impediments, refuse vaccination against COVID-19

☒ 0 ☐ 1 ☐ 2 ☐ 3 ☐ 4 ☐ 5 ☐ 6 ☐ 7 ☐ 8 ☐ 9 ☐ 10

It is preferable to adopt measures of social distancing and caution than imposing a legal obligation on COVID vaccination

☒ 0 ☐ 1 ☐ 2 ☐ 3 ☐ 4 ☐ 5 ☐ 6 ☐ 7 ☐ 8 ☐ 9 ☐ 10

Evidence on the efficacy and safety of COVID-19 vaccines is uncertain due to their rapid development

☒ 0 ☐ 1 ☐ 2 ☐ 3 ☐ 4 ☐ 5 ☐ 6 ☐ 7 ☐ 8 ☐ 9 ☐ 10

People who refuse to undergo COVID 19 vaccination and fall ill should bear the additional financial burden

☒ 0 ☐ 1 ☐ 2 ☐ 3 ☐ 4 ☐ 5 ☐ 6 ☐ 7 ☐ 8 ☐ 9 ☐ 10

Do you have or are you encouraging your family and / or friends to vaccinate against COVID-19?

☐ Yes

☐ No

☐ Yes, but only to the most fragile persons

What topic would you like to receive more information on? (It is possible to indicate more than one answer):

☐ Types of COVID-19 vaccines

☐ Effectiveness of COVID-19 vaccines

☐ Side effects and safety profile of COVID-19 vaccines

☐ I don't think I need to improve my knowledge

☐ Other

Specify other topics:
